# Giant Hyperkeratotic Lymphangioma Circumscriptum on the Thigh of a 79-Year-Old Woman: A Report of a Rare Case

**DOI:** 10.7759/cureus.79884

**Published:** 2025-03-01

**Authors:** Wael Aljehani, Shaheed Antlee, Reeman Aljohani, Taif Tharwat, Lujain Alrohaily, Badr Aljohani

**Affiliations:** 1 Department of Dermatology, King Salman bin Abdulaziz Medical City, Madinah, SAU; 2 Department of General Medicine, King Fahad Hospital, Madinah, SAU; 3 Department of Dermatology, Heraa General Hospital, Makkah, SAU; 4 Department of General Medicine, King Salman bin Abdulaziz Medical City, Madinah, SAU

**Keywords:** benign, case report, elderly, giant lesion, hamartomatous, hyperkeratotic, lymphangioma circumscriptum, lymphatic malformation

## Abstract

Lymphangioma circumscriptum (LC) is a rare benign lymphatic anomaly that often manifests during childhood. We report an atypical instance of enormous hyperkeratotic LC in an older patient, augmenting the little literature on severe manifestations of this illness. This is a case of a 79-year-old woman with a medical history notable for deep vein thrombosis (DVT) and morbid obesity who presented with a developing skin lesion on her left thigh. During the clinical examination, a well-defined, verrucous lesion measuring approximately 15 cm × 2 cm and resembling a cauliflower was identified. The excisional samples obtained for histological analysis to verify the diagnosis revealed an edematous fibro-adipose stroma with dilated lymphatic channels, hyperkeratosis, and moderate papillomatosis, confirming the diagnosis of hyperkeratotic LC. To our knowledge, this is the first reported case involving LC late in life, occurring in an unusual site with gigantic proportions and being hyperkeratotic, which are all regarded as atypical features for LC. The patient, having a history of DVT and being obese, would most likely have lymphatic dysfunction, predisposing her to the development of LC. The form of hyperkeratotic LC seen in this case is even less common than the more familiar presentation of LC, which typically forms large, translucent bullae. This case highlights the different clinical presentations of LC and emphasizes that it should be regarded as a differential diagnosis for verrucous lesions, especially in older people. It also emphasizes the significance of a biopsy in validating instances with unusual manifestations, such as in older age groups, bigger lesion sizes, and atypical sites. Further research is necessary to elucidate the etiopathogenetic causes of acquired LC in adults and develop therapeutic procedures for large lesions.

## Introduction

Lymphangioma circumscriptum (LC), also known as a microcystic lymphatic malformation, is a benign hamartomatous malformation of the lymphatic channels, leading to the dilation of lymphatic vessels in the skin and subcutaneous tissue [1,2]. The typical clinical appearance is a cluster of small, fluid-filled, septate vesicles that contain lymphatic fluid and resemble frogspawn. These lesions range in color from clear to pink, dark red, brown, or black and may become warty, especially on the palms or soles. LC can be found anywhere on the body, with a predilection for the proximal limbs because they have an extensive lymphatic network (e.g., axilla, shoulders, groins, buttocks, and perineum) [3]. There have also been reports of cases involving the tongue and vulva [4]. The lesion has been classified as either congenital, which appears in childhood, usually before the age of five, due to improper development of lymphatic channels with no connection to the main lymphatic drainage [5], or acquired, which manifests in adulthood because of surgery, infections, or radiation that disrupts a previously standard lymphatic system [6-8]. In this report, we describe a case of a giant LC measuring approximately 15 cm × 2 cm on the lateral side of the left thigh of a 79-year-old patient.

## Case presentation

A 79-year-old bedridden woman with a medical history of hypertension, hypothyroidism, morbid obesity, lung fibrosis, and bilateral lower limb deep vein thrombosis (DVT) on apixaban presented to the dermatology clinic with multiple chronic, progressive lesion groups on the lateral side of the left thigh. The lesions were painless, non-itchy, and odorless and had been present for the past year.

**Figure 1:**
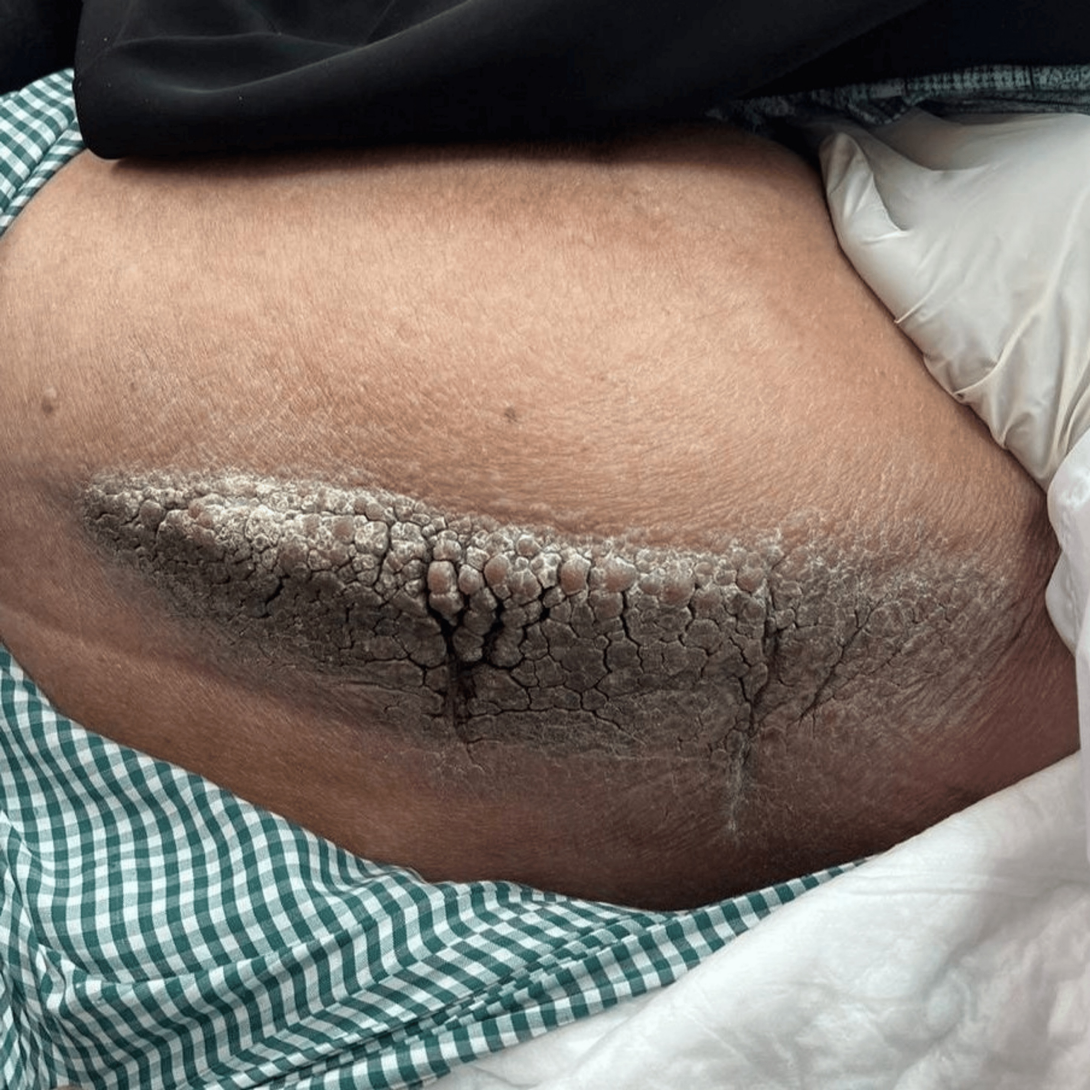
Lymphangioma circumscriptum. A single, well-demarcated, thickened hyperkeratotic plaque measuring approximately 15 × 7 cm is observed on the patient's left lateral thigh. The lesion exhibits a fissured and cracked appearance with thick scales, brown discoloration, and evident lichenification. There are no signs of purulent discharge or bleeding. It extends laterally without involving the surrounding tissue. The skin texture is rough and desquamating, indicative of chronicity.

On examination, a well-demarcated verrucous lesion measuring approximately 15 cm × 2 cm was noted on the lateral side of the left thigh, resembling a cauliflower. No other skin abnormalities were observed, and the hair, nails, and mucous membranes were unremarkable (Figure 1). Based on the clinical appearance, the primary differential diagnoses included hyperkeratotic LC, lymphangiectasis, molluscum contagiosum, warts, and epidermal nevi. The patient underwent two excisional biopsies under local anesthesia for further pathological evaluation. A fusidic acid ointment was prescribed for post-procedure care, and a comprehensive wound care plan was established. Gross examination revealed two skin specimens, one measuring 1.5 cm × 0.6 cm × 0.5 cm and 1.0 cm × 0.7 cm × 0.3 cm.

**Figure 2:**
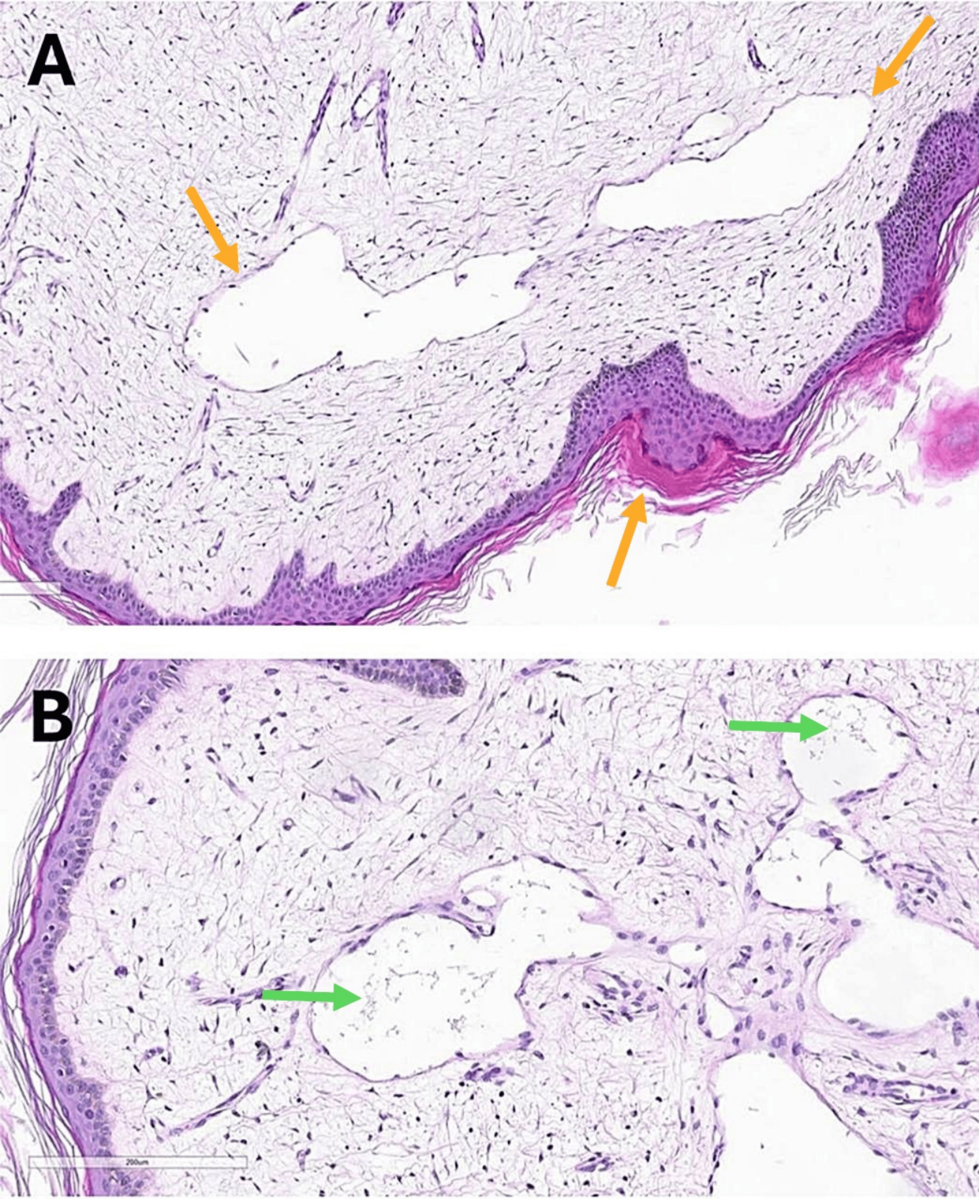
Histopathology of a skin punch biopsy from the patient’s thigh, stained with hematoxylin and eosin at 200× magnification. (A) The section shows hyperkeratosis and mild papillomatosis of the epidermis with dilated lymphatic channels in the dermis (orange arrows). (B) Thin-walled, dilated lymphatic channels in the dermis, some containing proteinaceous material within the lumen (green arrows).

Microscopic evaluation showed edematous fibro-adipose stroma, dilated lymphatic channels, hyperkeratosis, and mild papillomatosis, consistent with a benign fibroepithelial polyp, confirming the diagnosis of hyperkeratotic LC (Figures 2A, 2B). The patient was reassured about the benign nature of the condition and informed that effective treatment options were available. A complete surgical excision was advised as the optimal approach to remove the lesion and minimize the risk of recurrence. Post-surgical excision care, including proper wound management with surgical dressings, was emphasized. The patient was also counseled on the importance of regular follow-up visits for close monitoring, early detection of any recurrence, and additional management if necessary. Unfortunately, the patient was lost to follow-up, making long-term assessment and outcome evaluation challenging.

## Discussion

This case report adds new data to the literature on this rare dermatosis by presenting an exceptional case of giant hyperkeratotic LC on the thigh of an elderly patient. LC is typically a childhood lesion, with most cases diagnosed before the age of five. However, in our case, the lesion remained unrecognized until the patient was 79 years old, representing a less common acquired variant of LC. Other case reports of acquired LC in adults, which are commonly associated with previous surgery, radiation therapy, or infection, support the late-onset hypothesis [9,10]. Because her past medical history was significant for DVT and morbid obesity, these factors might have contributed to lymphatic dysfunction, which could have, in turn, led to the incitement of LC, as per the mechanisms proposed in the literature [11,12].

In our case, the size of the lesion (approximately 15 cm × 2 cm) is particularly remarkable. While LC lesions can vary in size, they are generally found to range from a few millimeters to several centimeters [10]. Lesions of such gigantic dimensions, as observed in our patient, are extremely rare, with only a few cases reported in the available literature [13,14]. This giant presentation stresses the potential for LC to grow massively, particularly in cases where delay occurs in its diagnosis and therapy.

The hyperkeratotic nature of the lesion is another exciting point in our case. Classically, LC presents as a translucent bulla; however, in our patient, the lesion exhibited a verrucous structure resembling a cauliflower. This hyperkeratotic variant has been described in the literature but is rarer than the classical variant [15,16]. Hyperkeratosis may be caused by chronic irritation and supposed lymph stagnation, giving rise to changes at the epidermal level over the years [17].

Histopathological findings in our case were like those reported earlier for LC, including dilated lymphatic channels, edematous stroma, and epidermal changes with hyperkeratosis and mild papillomatosis [13,18]. These features help confirm the diagnosis and reemphasize the vital role of histopathological examination in atypical presentations of LC.

LC management is complex, especially in giant lesions. The current case mandated complete excision only for investigative purposes; however, complete surgical excision is often the treatment of choice for symptomatic lesions or those that are cosmetically concerning. Due to its large size, complete excision may not be a feasible option, as noted in other reports documenting giant LC. Other treatment modalities that have shown promise and are worthy of consideration in case our patient pursues further intervention include sclerotherapy, laser therapy, and radiofrequency ablation. All these allow for the sparing of normal tissues and have therapeutic value in destroying abnormal tissues.

This report underscores the need to consider LC in diagnosing verrucous lesions, even in elderly patients. It further reemphasizes that early diagnosis and intervention could prevent giant lesions from forming, which can otherwise be challenging to manage. Future research should focus on determining the etiopathological factors leading to the development of acquired LC in adults, with an emphasis on the possible role of chronic venous insufficiency and obesity. Further long-term follow-up studies on cases of giant LC could shed much light on the natural history and optimal management strategies of this rare presentation.

## Conclusions

This case of giant hyperkeratotic LC on an atypical site of an elderly patient adds to the limited literature on extreme presentations of this condition. It emphasizes the diverse clinical manifestations of LC and the importance of histopathological confirmation in atypical cases. It is important to keep acquired LC in the differential diagnosis when evaluating adult patients with a new onset of asymptomatic vesicular lesions. Increased awareness of this condition can help prevent misdiagnosis and allow for appropriate treatment. Further studies are warranted to enhance our understanding of the pathogenesis, progression, and management of giant LC lesions in adults.
